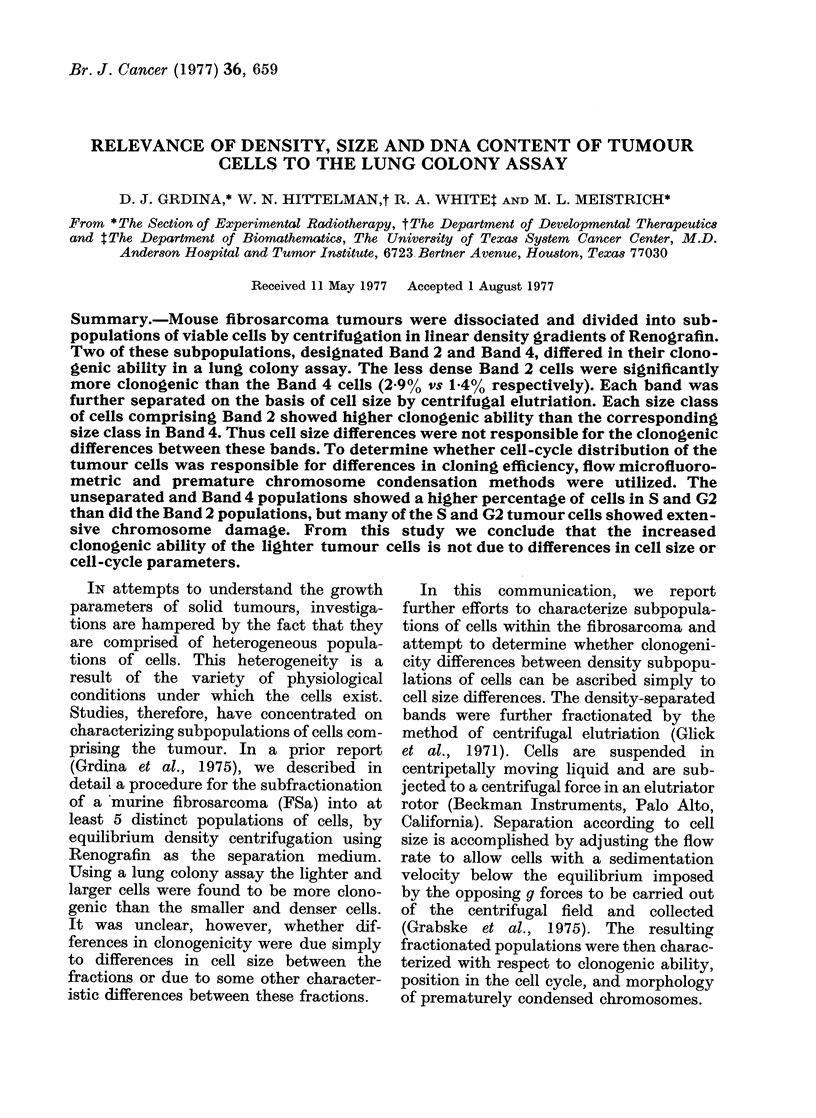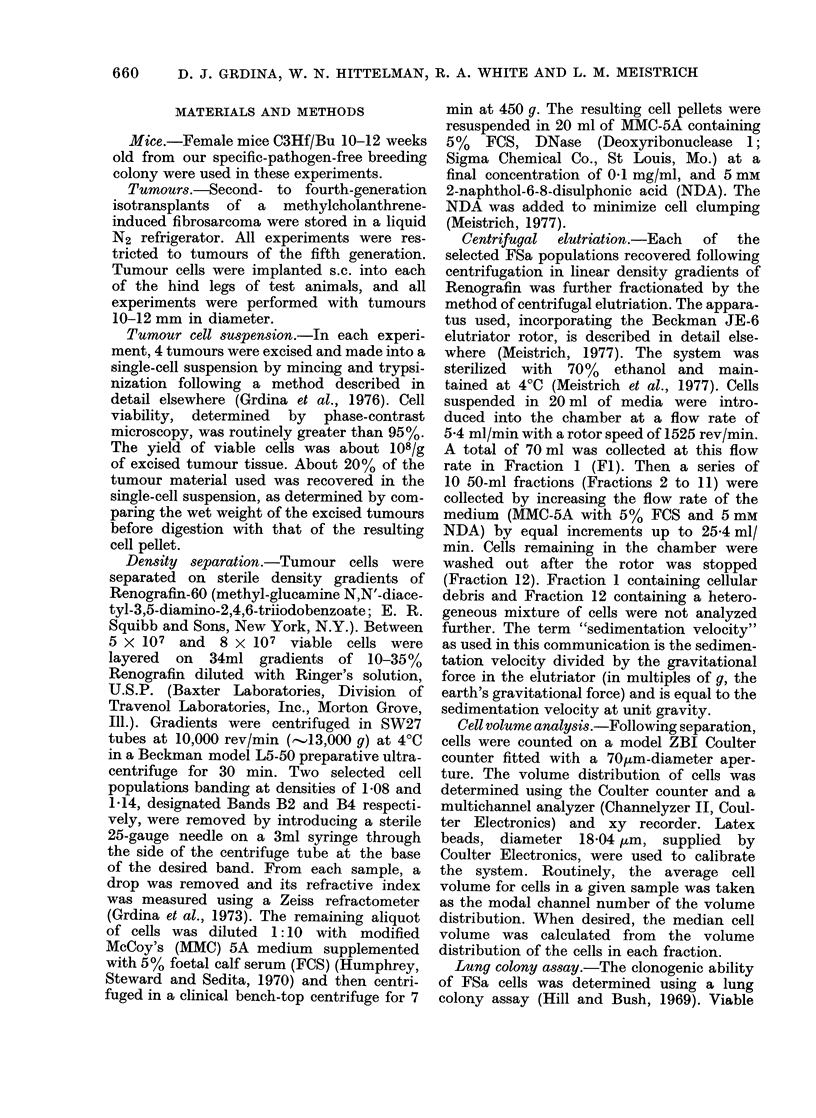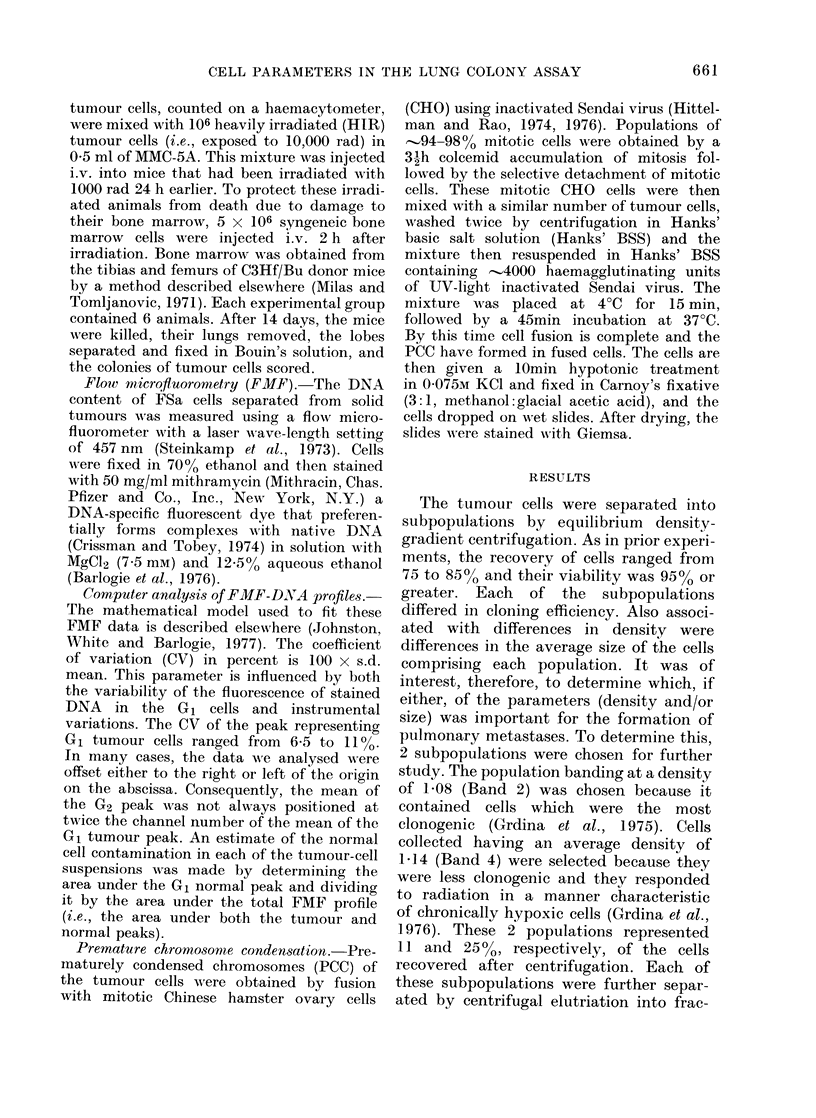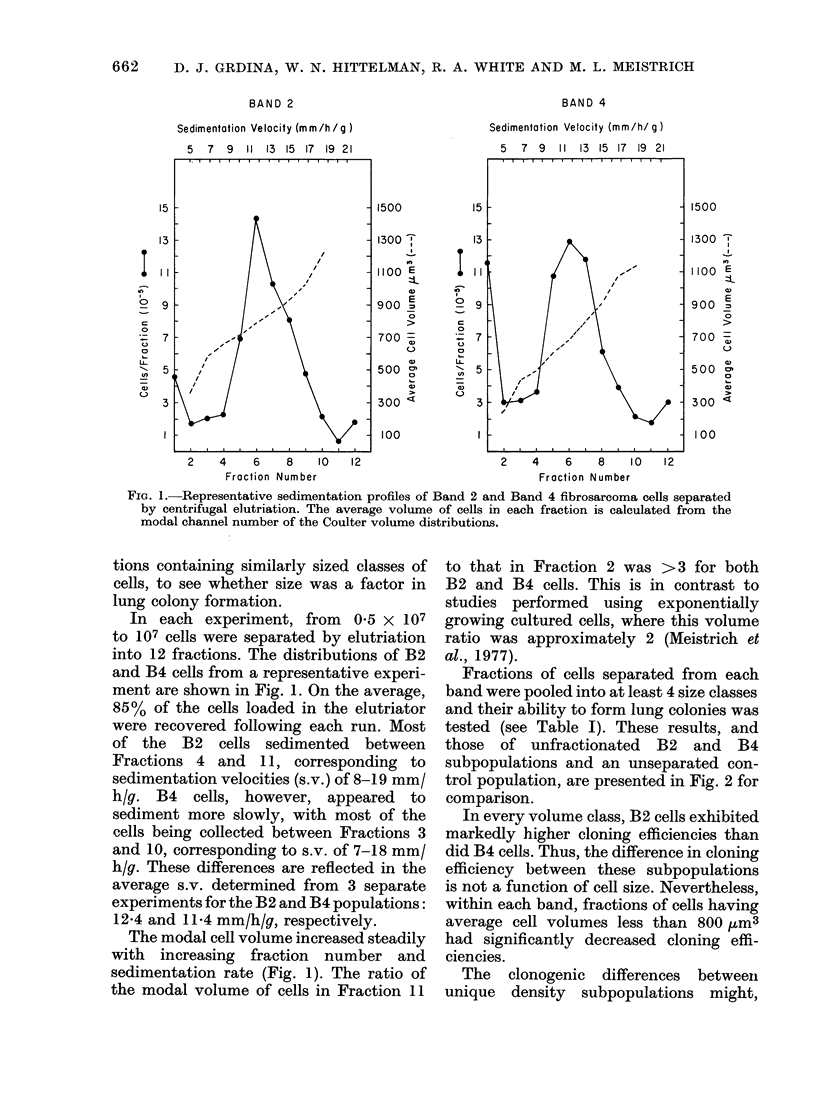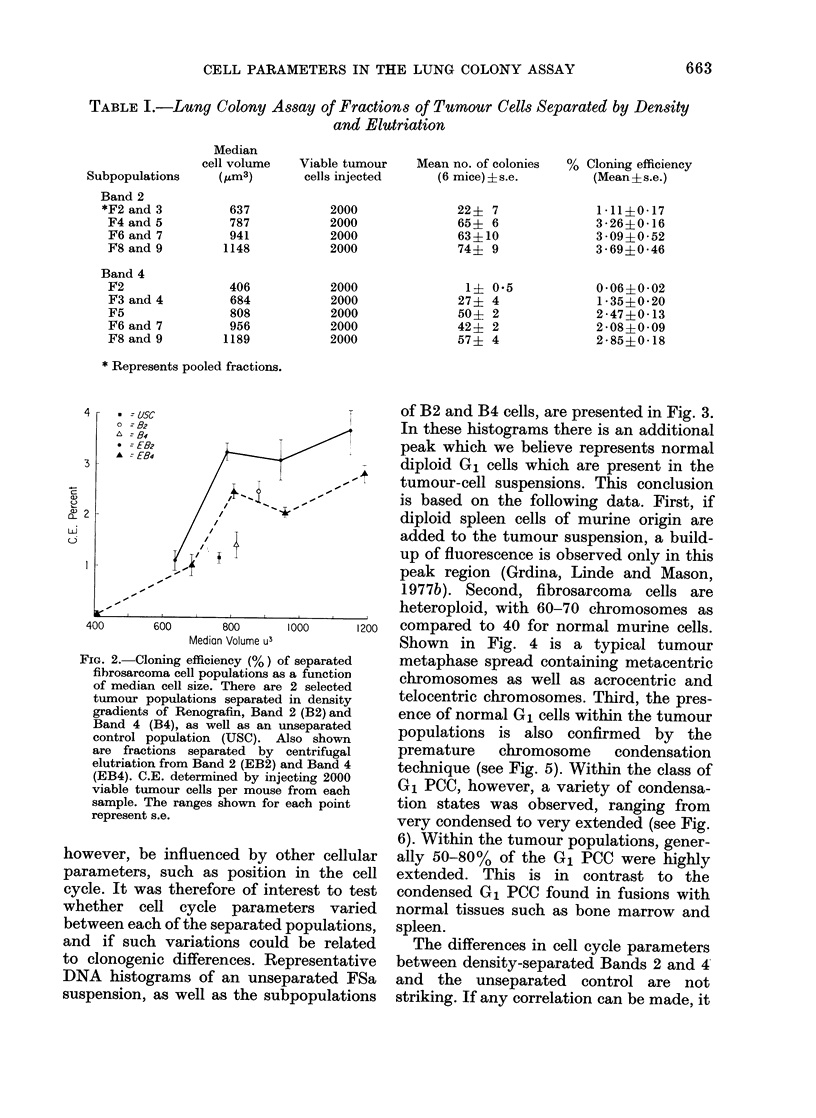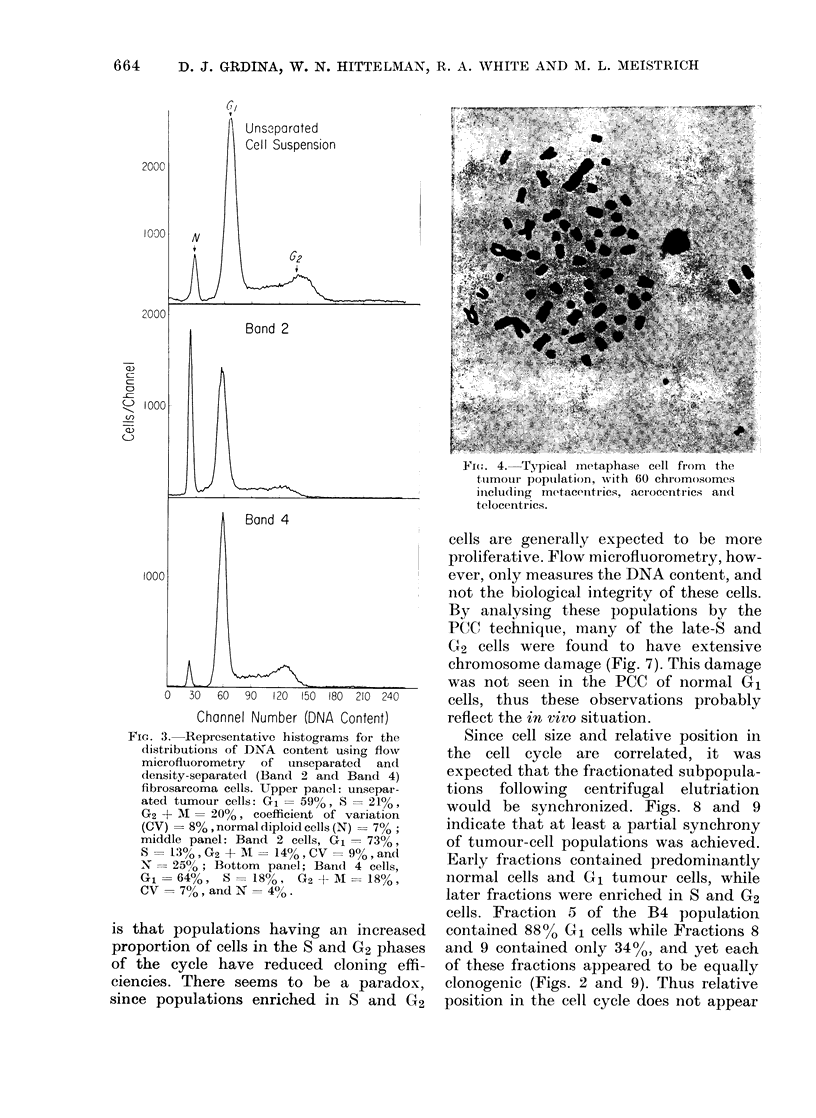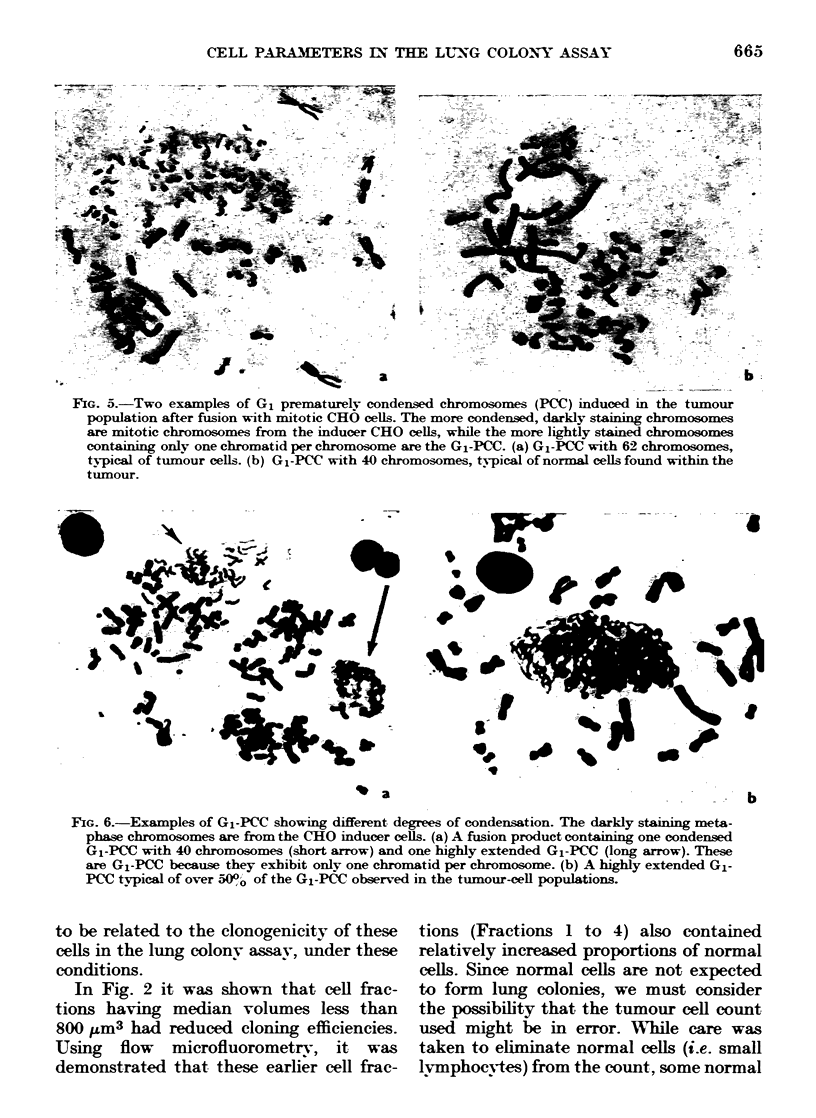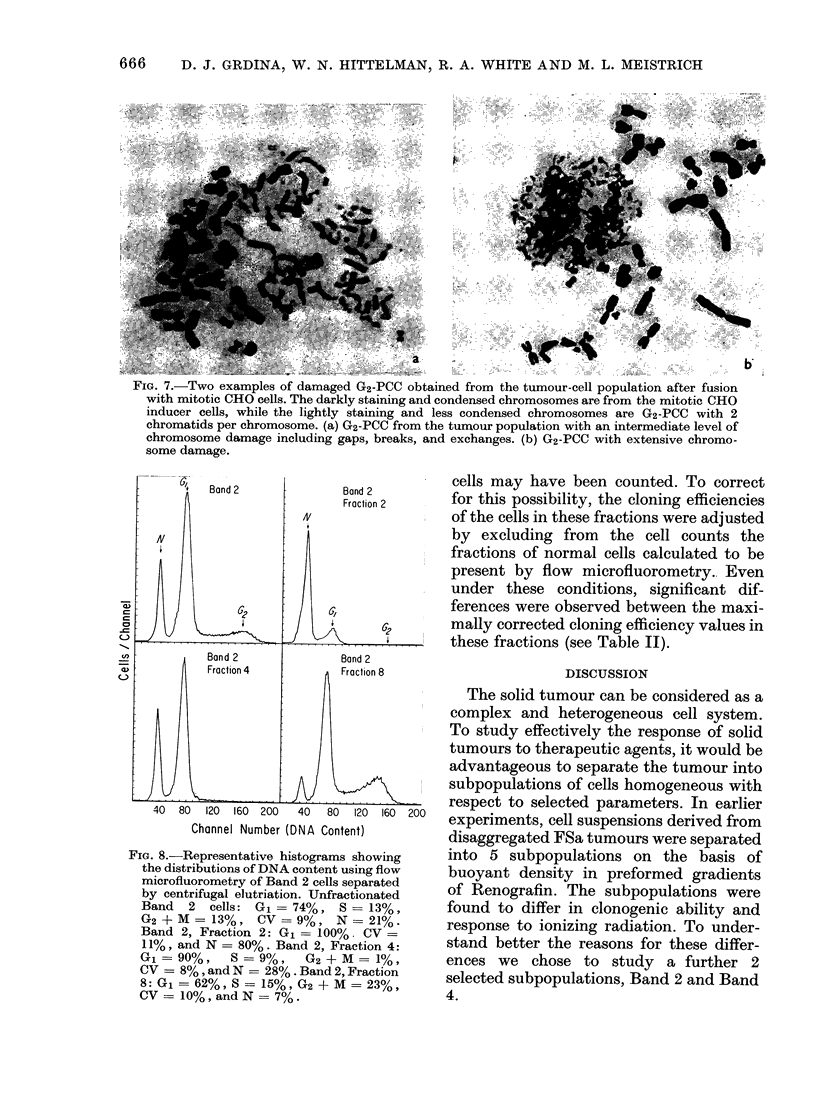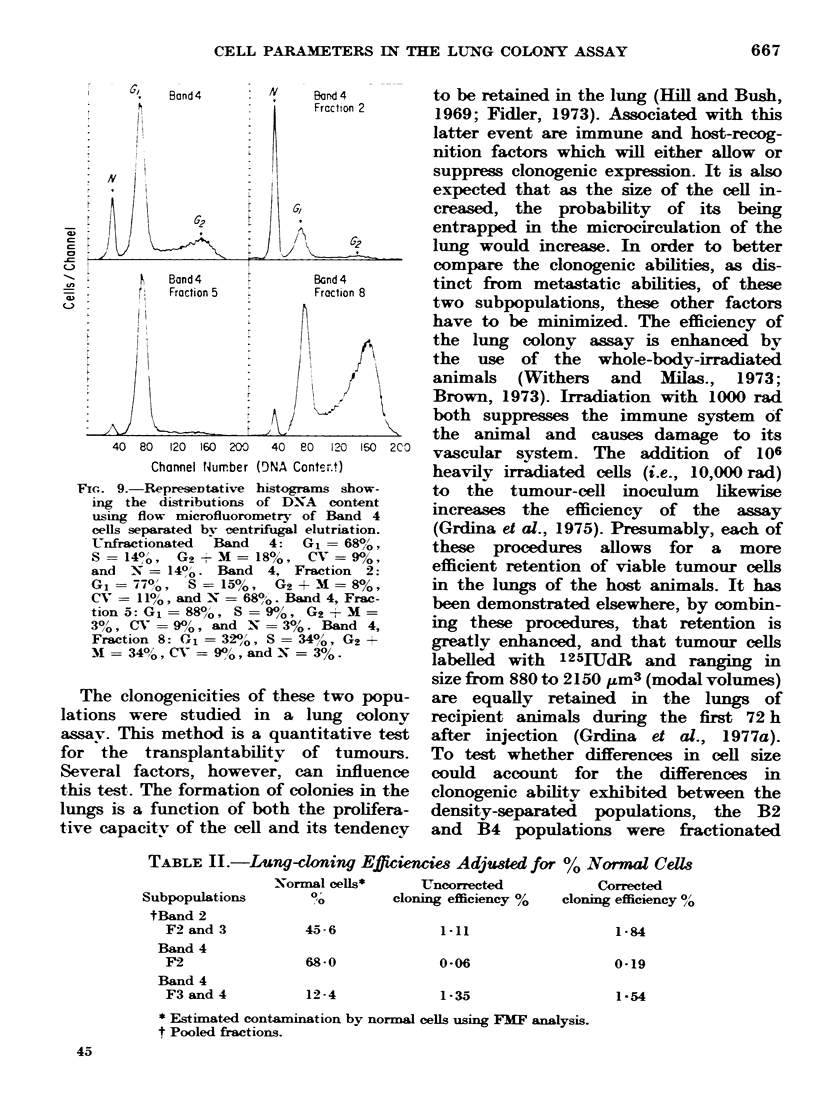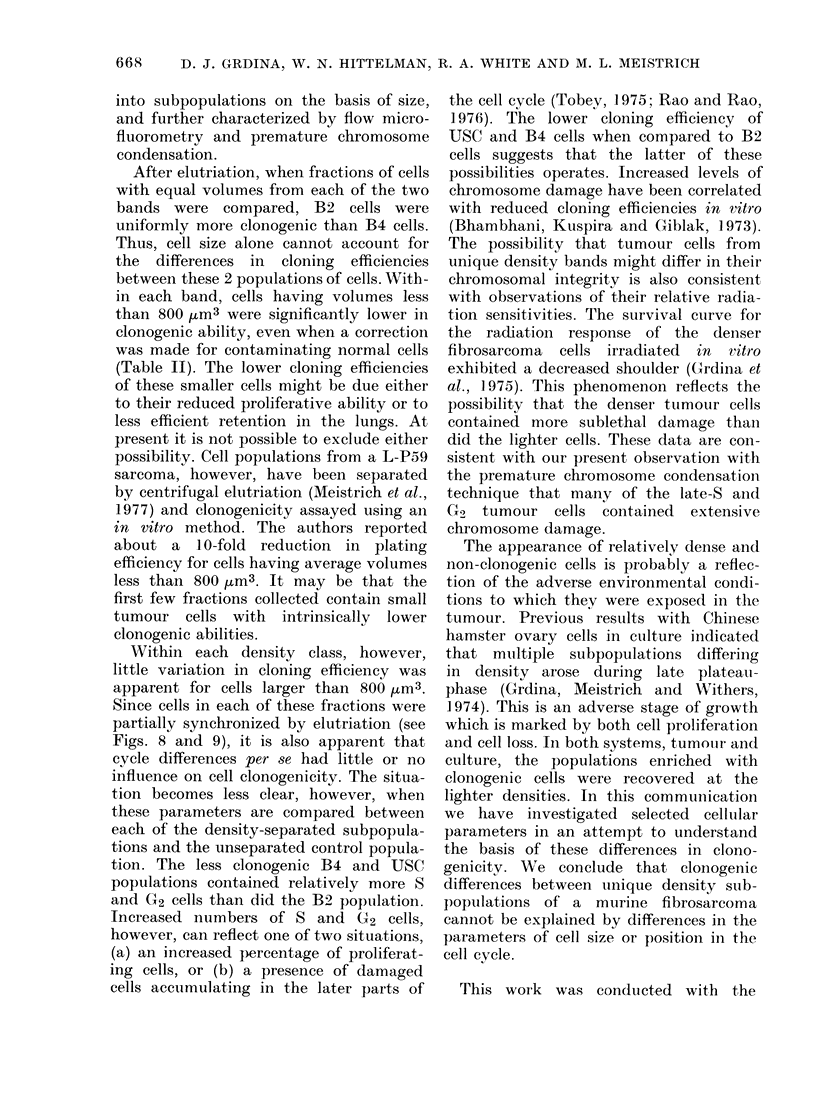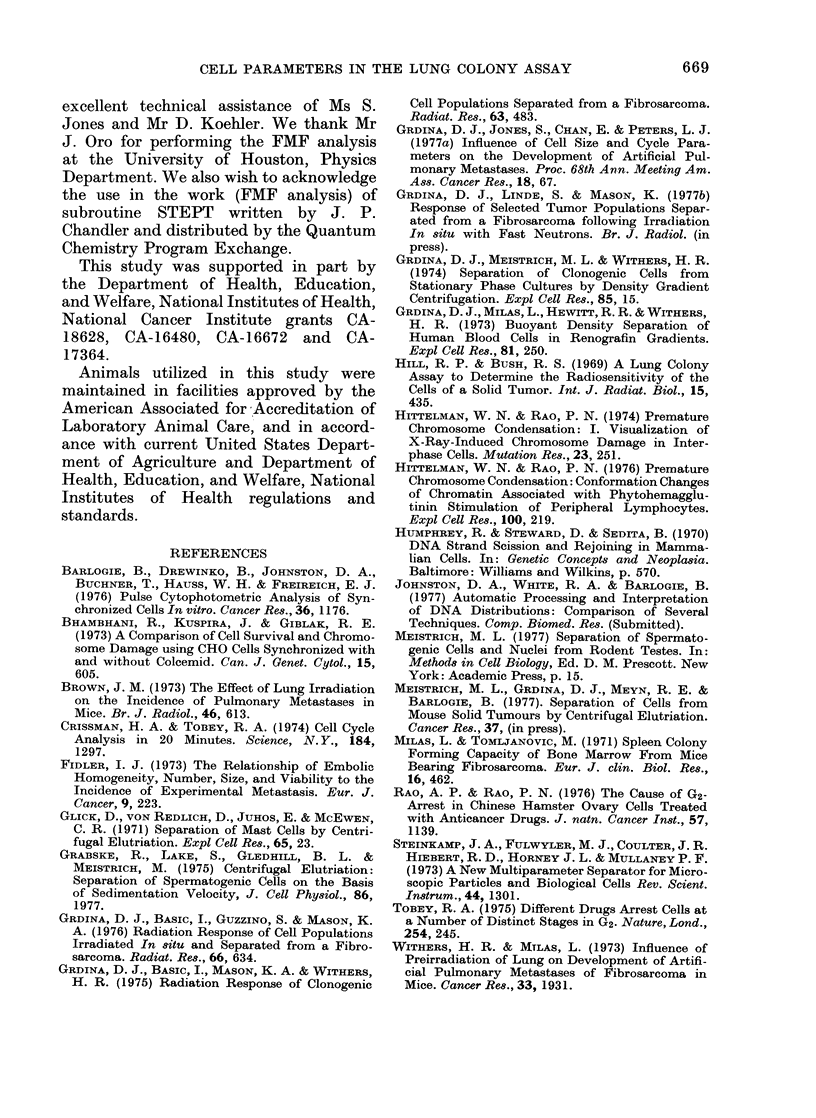# Relevance of density, size and DNA content of tumour cells to the lung colony assay.

**DOI:** 10.1038/bjc.1977.248

**Published:** 1977-12

**Authors:** D. J. Grdina, W. N. Hittelman, R. A. White, M. L. Meistrich

## Abstract

**Images:**


*Br. J. Cancer* (1977) **36**, 659

### RELEVANCE OF DENSITY, SIZE AND DNA CONTENT OF TUMOUR CELLS TO THE LUNG COLONY ASSAY

D. J. GRDINA,* W. N. HITTELMAN,† R. A. WHITE‡ AND M. L. MEISTRICH*

*From *The Section of Experimental Radiotherapy, †The Department of Developmental Therapeutics and ‡The Department of Biomathematics, The University of Texas System Cancer Center, M.D. Anderson Hospital and Tumor Institute,* 6723 *Bertner Avenue, Houston, Texas* 77030

Received 11 May 1977  Accepted 1 August 1977

**Summary.—Mouse fibrosarcoma tumours were dissociated and divided into subpopulations of viable cells by centrifugation in linear density gradients of Renografin. Two of these subpopulations, designated Band 2 and Band 4, differed in their clonogenic ability in a lung colony assay. The less dense Band 2 cells were significantly more clonogenic than the Band 4 cells (2·9% *vs* 1·4% respectively). Each band was further separated on the basis of cell size by centrifugal elutriation. Each size class of cells comprising Band 2 showed higher clonogenic ability than the corresponding size class in Band 4. Thus cell size differences were not responsible for the clonogenic differences between these bands. To determine whether cell-cycle distribution of the tumour cells was responsible for differences in cloning efficiency, flow microfluorometric and premature chromosome condensation methods were utilized. The unseparated and Band 4 populations showed a higher percentage of cells in S and G2 than did the Band 2 populations, but many of the S and G2 tumour cells showed extensive chromosome damage. From this study we conclude that the increased clonogenic ability of the lighter tumour cells is not due to differences in cell size or cell-cycle parameters.**

In attempts to understand the growth parameters of solid tumours, investigations are hampered by the fact that they are comprised of heterogeneous populations of cells. This heterogeneity is a result of the variety of physiological conditions under which the cells exist. Studies, therefore, have concentrated on characterizing subpopulations of cells comprising the tumour. In a prior report (Grdina *et al.*, 1975), we described in detail a procedure for the subfractionation of a murine fibrosarcoma (FSa) into at least 5 distinct populations of cells, by equilibrium density centrifugation using Renografin as the separation medium. Using a lung colony assay the lighter and larger cells were found to be more clonogenic than the smaller and denser cells. It was unclear, however, whether differences in clonogenicity were due simply to differences in cell size between the fractions or due to some other characteristic differences between these fractions.

In this communication, we report further efforts to characterize subpopulations of cells within the fibrosarcoma and attempt to determine whether clonogenicity differences between density subpopulations of cells can be ascribed simply to cell size differences. The density-separated bands were further fractionated by the method of centrifugal elutriation (Glick *et al.*, 1971). Cells are suspended in centripetally moving liquid and are subjected to a centrifugal force in an elutriator rotor (Beckman Instruments, Palo Alto, California). Separation according to cell size is accomplished by adjusting the flow rate to allow cells with a sedimentation velocity below the equilibrium imposed by the opposing *g* forces to be carried out of the centrifugal field and collected (Grabske *et al.*, 1975). The resulting fractionated populations were then characterized with respect to clonogenic ability, position in the cell cycle, and morphology of prematurely condensed chromosomes.

### MATERIALS AND METHODS

*Mice.*—Female mice C3Hf/Bu 10–12 weeks old from our specific-pathogen-free breeding colony were used in these experiments.

*Tumours.*—Second- to fourth-generation isotransplants of a methylcholanthrene-induced fibrosarcoma were stored in a liquid $N_2$ refrigerator. All experiments were restricted to tumours of the fifth generation. Tumour cells were implanted s.c. into each of the hind legs of test animals, and all experiments were performed with tumours 10–12 mm in diameter.

*Tumour cell suspension.*—In each experiment, 4 tumours were excised and made into a single-cell suspension by mincing and trypsinization following a method described in detail elsewhere (Grdina *et al.*, 1976). Cell viability, determined by phase-contrast microscopy, was routinely greater than 95%. The yield of viable cells was about $10^8$/g of excised tumour tissue. About 20% of the tumour material used was recovered in the single-cell suspension, as determined by comparing the wet weight of the excised tumours before digestion with that of the resulting cell pellet.

*Density separation.*—Tumour cells were separated on sterile density gradients of Renografin-60 (methyl-glucamine N,N′-diacetyl-3,5-diamino-2,4,6-triiodobenzoate; E. R. Squibb and Sons, New York, N.Y.). Between $5 \times 10^7$ and $8 \times 10^7$ viable cells were layered on 34ml gradients of 10–35% Renografin diluted with Ringer's solution, U.S.P. (Baxter Laboratories, Division of Travenol Laboratories, Inc., Morton Grove, Ill.). Gradients were centrifuged in SW27 tubes at 10,000 rev/min ($\sim$13,000 *g*) at 4°C in a Beckman model L5-50 preparative ultracentrifuge for 30 min. Two selected cell populations banding at densities of 1·08 and 1·14, designated Bands B2 and B4 respectively, were removed by introducing a sterile 25-gauge needle on a 3ml syringe through the side of the centrifuge tube at the base of the desired band. From each sample, a drop was removed and its refractive index was measured using a Zeiss refractometer (Grdina *et al.*, 1973). The remaining aliquot of cells was diluted 1:10 with modified McCoy's (MMC) 5A medium supplemented with 5% foetal calf serum (FCS) (Humphrey, Steward and Sedita, 1970) and then centrifuged in a clinical bench-top centrifuge for 7 min at 450 *g*. The resulting cell pellets were resuspended in 20 ml of MMC-5A containing 5% FCS, DNase (Deoxyribonuclease 1; Sigma Chemical Co., St Louis, Mo.) at a final concentration of 0·1 mg/ml, and 5 mM 2-naphthol-6-8-disulphonic acid (NDA). The NDA was added to minimize cell clumping (Meistrich, 1977).

*Centrifugal elutriation.*—Each of the selected FSa populations recovered following centrifugation in linear density gradients of Renografin was further fractionated by the method of centrifugal elutriation. The apparatus used, incorporating the Beckman JE-6 elutriator rotor, is described in detail elsewhere (Meistrich, 1977). The system was sterilized with 70% ethanol and maintained at 4°C (Meistrich *et al.*, 1977). Cells suspended in 20 ml of media were introduced into the chamber at a flow rate of 5·4 ml/min with a rotor speed of 1525 rev/min. A total of 70 ml was collected at this flow rate in Fraction 1 (F1). Then a series of 10 50-ml fractions (Fractions 2 to 11) were collected by increasing the flow rate of the medium (MMC-5A with 5% FCS and 5 mM NDA) by equal increments up to 25·4 ml/min. Cells remaining in the chamber were washed out after the rotor was stopped (Fraction 12). Fraction 1 containing cellular debris and Fraction 12 containing a heterogeneous mixture of cells were not analyzed further. The term "sedimentation velocity" as used in this communication is the sedimentation velocity divided by the gravitational force in the elutriator (in multiples of *g*, the earth's gravitational force) and is equal to the sedimentation velocity at unit gravity.

*Cell volume analysis.*—Following separation, cells were counted on a model ZBI Coulter counter fitted with a 70$\mu$m-diameter aperture. The volume distribution of cells was determined using the Coulter counter and a multichannel analyzer (Channelyzer II, Coulter Electronics) and xy recorder. Latex beads, diameter 18·04 $\mu$m, supplied by Coulter Electronics, were used to calibrate the system. Routinely, the average cell volume for cells in a given sample was taken as the modal channel number of the volume distribution. When desired, the median cell volume was calculated from the volume distribution of the cells in each fraction.

*Lung colony assay.*—The clonogenic ability of FSa cells was determined using a lung colony assay (Hill and Bush, 1969). Viable tumour cells, counted on a haemacytometer, were mixed with $10^6$ heavily irradiated (HIR) tumour cells (*i.e.*, exposed to 10,000 rad) in 0·5 ml of MMC-5A. This mixture was injected i.v. into mice that had been irradiated with 1000 rad 24 h earlier. To protect these irradiated animals from death due to damage to their bone marrow, $5 \times 10^6$ syngeneic bone marrow cells were injected i.v. 2 h after irradiation. Bone marrow was obtained from the tibias and femurs of C3Hf/Bu donor mice by a method described elsewhere (Milas and Tomljanovic, 1971). Each experimental group contained 6 animals. After 14 days, the mice were killed, their lungs removed, the lobes separated and fixed in Bouin's solution, and the colonies of tumour cells scored.

*Flow microfluorometry* (*FMF*).—The DNA content of FSa cells separated from solid tumours was measured using a flow microfluorometer with a laser wave-length setting of 457 nm (Steinkamp *et al.*, 1973). Cells were fixed in 70% ethanol and then stained with 50 mg/ml mithramycin (Mithracin, Chas. Pfizer and Co., Inc., New York, N.Y.) a DNA-specific fluorescent dye that preferentially forms complexes with native DNA (Crissman and Tobey, 1974) in solution with $MgCl_2$ (7·5 mM) and 12·5% aqueous ethanol (Barlogie *et al.*, 1976).

*Computer analysis of FMF-DNA profiles.*—The mathematical model used to fit these FMF data is described elsewhere (Johnston, White and Barlogie, 1977). The coefficient of variation (CV) in percent is 100 × s.d. mean. This parameter is influenced by both the variability of the fluorescence of stained DNA in the $G_1$ cells and instrumental variations. The CV of the peak representing $G_1$ tumour cells ranged from 6·5 to 11%. In many cases, the data we analysed were offset either to the right or left of the origin on the abscissa. Consequently, the mean of the $G_2$ peak was not always positioned at twice the channel number of the mean of the $G_1$ tumour peak. An estimate of the normal cell contamination in each of the tumour-cell suspensions was made by determining the area under the $G_1$ normal peak and dividing it by the area under the total FMF profile (*i.e.*, the area under both the tumour and normal peaks).

*Premature chromosome condensation.*—Prematurely condensed chromosomes (PCC) of the tumour cells were obtained by fusion with mitotic Chinese hamster ovary cells (CHO) using inactivated Sendai virus (Hittelman and Rao, 1974, 1976). Populations of ~94–98% mitotic cells were obtained by a 3½h colcemid accumulation of mitosis followed by the selective detachment of mitotic cells. These mitotic CHO cells were then mixed with a similar number of tumour cells, washed twice by centrifugation in Hanks' basic salt solution (Hanks' BSS) and the mixture then resuspended in Hanks' BSS containing ~4000 haemagglutinating units of UV-light inactivated Sendai virus. The mixture was placed at 4°C for 15 min, followed by a 45min incubation at 37°C. By this time cell fusion is complete and the PCC have formed in fused cells. The cells are then given a 10min hypotonic treatment in 0·075M KCl and fixed in Carnoy's fixative (3:1, methanol:glacial acetic acid), and the cells dropped on wet slides. After drying, the slides were stained with Giemsa.

### RESULTS

The tumour cells were separated into subpopulations by equilibrium density-gradient centrifugation. As in prior experiments, the recovery of cells ranged from 75 to 85% and their viability was 95% or greater. Each of the subpopulations differed in cloning efficiency. Also associated with differences in density were differences in the average size of the cells comprising each population. It was of interest, therefore, to determine which, if either, of the parameters (density and/or size) was important for the formation of pulmonary metastases. To determine this, 2 subpopulations were chosen for further study. The population banding at a density of 1·08 (Band 2) was chosen because it contained cells which were the most clonogenic (Grdina *et al.*, 1975). Cells collected having an average density of 1·14 (Band 4) were selected because they were less clonogenic and they responded to radiation in a manner characteristic of chronically hypoxic cells (Grdina *et al.*, 1976). These 2 populations represented 11 and 25%, respectively, of the cells recovered after centrifugation. Each of these subpopulations were further separated by centrifugal elutriation into frac-

FIG. 1.—Representative sedimentation profiles of Band 2 and Band 4 fibrosarcoma cells separated by centrifugal elutriation. The average volume of cells in each fraction is calculated from the modal channel number of the Coulter volume distributions.

tions containing similarly sized classes of cells, to see whether size was a factor in lung colony formation.

In each experiment, from $0{\cdot}5 \times 10^7$ to $10^7$ cells were separated by elutriation into 12 fractions. The distributions of B2 and B4 cells from a representative experiment are shown in Fig. 1. On the average, 85% of the cells loaded in the elutriator were recovered following each run. Most of the B2 cells sedimented between Fractions 4 and 11, corresponding to sedimentation velocities (s.v.) of 8–19 mm/h/*g*. B4 cells, however, appeared to sediment more slowly, with most of the cells being collected between Fractions 3 and 10, corresponding to s.v. of 7–18 mm/h/*g*. These differences are reflected in the average s.v. determined from 3 separate experiments for the B2 and B4 populations: 12·4 and 11·4 mm/h/*g*, respectively.

The modal cell volume increased steadily with increasing fraction number and sedimentation rate (Fig. 1). The ratio of the modal volume of cells in Fraction 11 to that in Fraction 2 was >3 for both B2 and B4 cells. This is in contrast to studies performed using exponentially growing cultured cells, where this volume ratio was approximately 2 (Meistrich *et al.*, 1977).

Fractions of cells separated from each band were pooled into at least 4 size classes and their ability to form lung colonies was tested (see Table I). These results, and those of unfractionated B2 and B4 subpopulations and an unseparated control population, are presented in Fig. 2 for comparison.

In every volume class, B2 cells exhibited markedly higher cloning efficiencies than did B4 cells. Thus, the difference in cloning efficiency between these subpopulations is not a function of cell size. Nevertheless, within each band, fractions of cells having average cell volumes less than 800 $\mu m^3$ had significantly decreased cloning efficiencies.

The clonogenic differences between unique density subpopulations might,

TABLE I.—*Lung Colony Assay of Fractions of Tumour Cells Separated by Density and Elutriation*

| Subpopulations | Median cell volume ($\mu m^3$) | Viable tumour cells injected | Mean no. of colonies (6 mice) ± s.e. | % Cloning efficiency (Mean ± s.e.) |
|---|---|---|---|---|
| Band 2 | | | | |
| *F2 and 3 | 637 | 2000 | 22 ± 7 | 1·11 ± 0·17 |
| F4 and 5 | 787 | 2000 | 65 ± 6 | 3·26 ± 0·16 |
| F6 and 7 | 941 | 2000 | 63 ± 10 | 3·09 ± 0·52 |
| F8 and 9 | 1148 | 2000 | 74 ± 9 | 3·69 ± 0·46 |
| Band 4 | | | | |
| F2 | 406 | 2000 | 1 ± 0·5 | 0·06 ± 0·02 |
| F3 and 4 | 684 | 2000 | 27 ± 4 | 1·35 ± 0·20 |
| F5 | 808 | 2000 | 50 ± 2 | 2·47 ± 0·13 |
| F6 and 7 | 956 | 2000 | 42 ± 2 | 2·08 ± 0·09 |
| F8 and 9 | 1189 | 2000 | 57 ± 4 | 2·85 ± 0·18 |

* Represents pooled fractions.

FIG. 2.—Cloning efficiency (%) of separated fibrosarcoma cell populations as a function of median cell size. There are 2 selected tumour populations separated in density gradients of Renografin, Band 2 (B2) and Band 4 (B4), as well as an unseparated control population (USC). Also shown are fractions separated by centrifugal elutriation from Band 2 (EB2) and Band 4 (EB4). C.E. determined by injecting 2000 viable tumour cells per mouse from each sample. The ranges shown for each point represent s.e.

however, be influenced by other cellular parameters, such as position in the cell cycle. It was therefore of interest to test whether cell cycle parameters varied between each of the separated populations, and if such variations could be related to clonogenic differences. Representative DNA histograms of an unseparated FSa suspension, as well as the subpopulations of B2 and B4 cells, are presented in Fig. 3. In these histograms there is an additional peak which we believe represents normal diploid $G_1$ cells which are present in the tumour-cell suspensions. This conclusion is based on the following data. First, if diploid spleen cells of murine origin are added to the tumour suspension, a build-up of fluorescence is observed only in this peak region (Grdina, Linde and Mason, 1977*b*). Second, fibrosarcoma cells are heteroploid, with 60–70 chromosomes as compared to 40 for normal murine cells. Shown in Fig. 4 is a typical tumour metaphase spread containing metacentric chromosomes as well as acrocentric and telocentric chromosomes. Third, the presence of normal $G_1$ cells within the tumour populations is also confirmed by the premature chromosome condensation technique (see Fig. 5). Within the class of $G_1$ PCC, however, a variety of condensation states was observed, ranging from very condensed to very extended (see Fig. 6). Within the tumour populations, generally 50–80% of the $G_1$ PCC were highly extended. This is in contrast to the condensed $G_1$ PCC found in fusions with normal tissues such as bone marrow and spleen.

The differences in cell cycle parameters between density-separated Bands 2 and 4 and the unseparated control are not striking. If any correlation can be made, it

FIG. 3.—Representative histograms for the distributions of DNA content using flow microfluorometry of unseparated and density-separated (Band 2 and Band 4) fibrosarcoma cells. Upper panel: unseparated tumour cells: $G_1 = 59\%$, $S = 21\%$, $G_2 + M = 20\%$, coefficient of variation (CV) $= 8\%$, normal diploid cells (N) $= 7\%$; middle panel: Band 2 cells, $G_1 = 73\%$, $S = 13\%$, $G_2 + M = 14\%$, $CV = 9\%$, and $N = 25\%$; Bottom panel; Band 4 cells, $G_1 = 64\%$, $S = 18\%$, $G_2 + M = 18\%$, $CV = 7\%$, and $N = 4\%$.

is that populations having an increased proportion of cells in the S and $G_2$ phases of the cycle have reduced cloning efficiencies. There seems to be a paradox, since populations enriched in S and $G_2$

FIG. 4.—Typical metaphase cell from the tumour population, with 60 chromosomes including metacentrics, acrocentrics and telocentrics.

cells are generally expected to be more proliferative. Flow microfluorometry, however, only measures the DNA content, and not the biological integrity of these cells. By analysing these populations by the PCC technique, many of the late-S and $G_2$ cells were found to have extensive chromosome damage (Fig. 7). This damage was not seen in the PCC of normal $G_1$ cells, thus these observations probably reflect the *in vivo* situation.

Since cell size and relative position in the cell cycle are correlated, it was expected that the fractionated subpopulations following centrifugal elutriation would be synchronized. Figs. 8 and 9 indicate that at least a partial synchrony of tumour-cell populations was achieved. Early fractions contained predominantly normal cells and $G_1$ tumour cells, while later fractions were enriched in S and $G_2$ cells. Fraction 5 of the B4 population contained 88% $G_1$ cells while Fractions 8 and 9 contained only 34%, and yet each of these fractions appeared to be equally clonogenic (Figs. 2 and 9). Thus relative position in the cell cycle does not appear

FIG. 5.—Two examples of $G_1$ prematurely condensed chromosomes (PCC) induced in the tumour population after fusion with mitotic CHO cells. The more condensed, darkly staining chromosomes are mitotic chromosomes from the inducer CHO cells, while the more lightly stained chromosomes containing only one chromatid per chromosome are the $G_1$-PCC. (a) $G_1$-PCC with 62 chromosomes, typical of tumour cells. (b) $G_1$-PCC with 40 chromosomes, typical of normal cells found within the tumour.

FIG. 6.—Examples of $G_1$-PCC showing different degrees of condensation. The darkly staining metaphase chromosomes are from the CHO inducer cells. (a) A fusion product containing one condensed $G_1$-PCC with 40 chromosomes (short arrow) and one highly extended $G_1$-PCC (long arrow). These are $G_1$-PCC because they exhibit only one chromatid per chromosome. (b) A highly extended $G_1$-PCC typical of over 50% of the $G_1$-PCC observed in the tumour-cell populations.

to be related to the clonogenicity of these cells in the lung colony assay, under these conditions.

In Fig. 2 it was shown that cell fractions having median volumes less than 800 $\mu m^3$ had reduced cloning efficiencies. Using flow microfluorometry, it was demonstrated that these earlier cell fractions (Fractions 1 to 4) also contained relatively increased proportions of normal cells. Since normal cells are not expected to form lung colonies, we must consider the possibility that the tumour cell count used might be in error. While care was taken to eliminate normal cells (*i.e.* small lymphocytes) from the count, some normal

FIG. 7.—Two examples of damaged $G_2$-PCC obtained from the tumour-cell population after fusion with mitotic CHO cells. The darkly staining and condensed chromosomes are from the mitotic CHO inducer cells, while the lightly staining and less condensed chromosomes are $G_2$-PCC with 2 chromatids per chromosome. (a) $G_2$-PCC from the tumour population with an intermediate level of chromosome damage including gaps, breaks, and exchanges. (b) $G_2$-PCC with extensive chromosome damage.

FIG. 8.—Representative histograms showing the distributions of DNA content using flow microfluorometry of Band 2 cells separated by centrifugal elutriation. Unfractionated Band 2 cells: $G_1 = 74\%$, $S = 13\%$, $G_2 + M = 13\%$, $CV = 9\%$, $N = 21\%$. Band 2, Fraction 2: $G_1 = 100\%$. $CV = 11\%$, and $N = 80\%$. Band 2, Fraction 4: $G_1 = 90\%$, $S = 9\%$, $G_2 + M = 1\%$, $CV = 8\%$, and $N = 28\%$. Band 2, Fraction 8: $G_1 = 62\%$, $S = 15\%$, $G_2 + M = 23\%$, $CV = 10\%$, and $N = 7\%$.

cells may have been counted. To correct for this possibility, the cloning efficiencies of the cells in these fractions were adjusted by excluding from the cell counts the fractions of normal cells calculated to be present by flow microfluorometry. Even under these conditions, significant differences were observed between the maximally corrected cloning efficiency values in these fractions (see Table II).

### DISCUSSION

The solid tumour can be considered as a complex and heterogeneous cell system. To study effectively the response of solid tumours to therapeutic agents, it would be advantageous to separate the tumour into subpopulations of cells homogeneous with respect to selected parameters. In earlier experiments, cell suspensions derived from disaggregated FSa tumours were separated into 5 subpopulations on the basis of buoyant density in preformed gradients of Renografin. The subpopulations were found to differ in clonogenic ability and response to ionizing radiation. To understand better the reasons for these differences we chose to study a further 2 selected subpopulations, Band 2 and Band 4.

FIG. 9.—Representative histograms showing the distributions of DNA content using flow microfluorometry of Band 4 cells separated by centrifugal elutriation. Unfractionated Band 4: $G_1 = 68\%$, $S = 14\%$, $G_2 + M = 18\%$, $CV = 9\%$, and $N = 14\%$. Band 4, Fraction 2: $G_1 = 77\%$, $S = 15\%$, $G_2 + M = 8\%$, $CV = 11\%$, and $N = 68\%$. Band 4, Fraction 5: $G_1 = 88\%$, $S = 9\%$, $G_2 + M = 3\%$, $CV = 9\%$, and $N = 3\%$. Band 4, Fraction 8: $G_1 = 32\%$, $S = 34\%$, $G_2 + M = 34\%$, $CV = 9\%$, and $N = 3\%$.

The clonogenicities of these two populations were studied in a lung colony assay. This method is a quantitative test for the transplantability of tumours. Several factors, however, can influence this test. The formation of colonies in the lungs is a function of both the proliferative capacity of the cell and its tendency to be retained in the lung (Hill and Bush, 1969; Fidler, 1973). Associated with this latter event are immune and host-recognition factors which will either allow or suppress clonogenic expression. It is also expected that as the size of the cell increased, the probability of its being entrapped in the microcirculation of the lung would increase. In order to better compare the clonogenic abilities, as distinct from metastatic abilities, of these two subpopulations, these other factors have to be minimized. The efficiency of the lung colony assay is enhanced by the use of the whole-body-irradiated animals (Withers and Milas., 1973; Brown, 1973). Irradiation with 1000 rad both suppresses the immune system of the animal and causes damage to its vascular system. The addition of $10^6$ heavily irradiated cells (*i.e.*, 10,000 rad) to the tumour-cell inoculum likewise increases the efficiency of the assay (Grdina *et al.*, 1975). Presumably, each of these procedures allows for a more efficient retention of viable tumour cells in the lungs of the host animals. It has been demonstrated elsewhere, by combining these procedures, that retention is greatly enhanced, and that tumour cells labelled with $^{125}$IUdR and ranging in size from 880 to 2150 $\mu m^3$ (modal volumes) are equally retained in the lungs of recipient animals during the first 72 h after injection (Grdina *et al.*, 1977*a*). To test whether differences in cell size could account for the differences in clonogenic ability exhibited between the density-separated populations, the B2 and B4 populations were fractionated

TABLE II.—*Lung-cloning Efficiencies Adjusted for % Normal Cells*

| Subpopulations | Normal cells* % | Uncorrected cloning efficiency % | Corrected cloning efficiency % |
|---|---|---|---|
| †Band 2 | | | |
| F2 and 3 | 45·6 | 1·11 | 1·84 |
| Band 4 | | | |
| F2 | 68·0 | 0·06 | 0·19 |
| Band 4 | | | |
| F3 and 4 | 12·4 | 1·35 | 1·54 |

* Estimated contamination by normal cells using FMF analysis.
† Pooled fractions.

into subpopulations on the basis of size, and further characterized by flow microfluorometry and premature chromosome condensation.

After elutriation, when fractions of cells with equal volumes from each of the two bands were compared, B2 cells were uniformly more clonogenic than B4 cells. Thus, cell size alone cannot account for the differences in cloning efficiencies between these 2 populations of cells. Within each band, cells having volumes less than 800 $\mu m^3$ were significantly lower in clonogenic ability, even when a correction was made for contaminating normal cells (Table II). The lower cloning efficiencies of these smaller cells might be due either to their reduced proliferative ability or to less efficient retention in the lungs. At present it is not possible to exclude either possibility. Cell populations from a L-P59 sarcoma, however, have been separated by centrifugal elutriation (Meistrich *et al.*, 1977) and clonogenicity assayed using an *in vitro* method. The authors reported about a 10-fold reduction in plating efficiency for cells having average volumes less than 800 $\mu m^3$. It may be that the first few fractions collected contain small tumour cells with intrinsically lower clonogenic abilities.

Within each density class, however, little variation in cloning efficiency was apparent for cells larger than 800 $\mu m^3$. Since cells in each of these fractions were partially synchronized by elutriation (see Figs. 8 and 9), it is also apparent that cycle differences *per se* had little or no influence on cell clonogenicity. The situation becomes less clear, however, when these parameters are compared between each of the density-separated subpopulations and the unseparated control population. The less clonogenic B4 and USC populations contained relatively more S and $G_2$ cells than did the B2 population. Increased numbers of S and $G_2$ cells, however, can reflect one of two situations, (a) an increased percentage of proliferating cells, or (b) a presence of damaged cells accumulating in the later parts of the cell cycle (Tobey, 1975; Rao and Rao, 1976). The lower cloning efficiency of USC and B4 cells when compared to B2 cells suggests that the latter of these possibilities operates. Increased levels of chromosome damage have been correlated with reduced cloning efficiencies *in vitro* (Bhambhani, Kuspira and Giblak, 1973). The possibility that tumour cells from unique density bands might differ in their chromosomal integrity is also consistent with observations of their relative radiation sensitivities. The survival curve for the radiation response of the denser fibrosarcoma cells irradiated *in vitro* exhibited a decreased shoulder (Grdina *et al.*, 1975). This phenomenon reflects the possibility that the denser tumour cells contained more sublethal damage than did the lighter cells. These data are consistent with our present observation with the premature chromosome condensation technique that many of the late-S and $G_2$ tumour cells contained extensive chromosome damage.

The appearance of relatively dense and non-clonogenic cells is probably a reflection of the adverse environmental conditions to which they were exposed in the tumour. Previous results with Chinese hamster ovary cells in culture indicated that multiple subpopulations differing in density arose during late plateau-phase (Grdina, Meistrich and Withers, 1974). This is an adverse stage of growth which is marked by both cell proliferation and cell loss. In both systems, tumour and culture, the populations enriched with clonogenic cells were recovered at the lighter densities. In this communication we have investigated selected cellular parameters in an attempt to understand the basis of these differences in clonogenicity. We conclude that clonogenic differences between unique density subpopulations of a murine fibrosarcoma cannot be explained by differences in the parameters of cell size or position in the cell cycle.

This work was conducted with the excellent technical assistance of Ms S. Jones and Mr D. Koehler. We thank Mr J. Oro for performing the FMF analysis at the University of Houston, Physics Department. We also wish to acknowledge the use in the work (FMF analysis) of subroutine STEPT written by J. P. Chandler and distributed by the Quantum Chemistry Program Exchange.

This study was supported in part by the Department of Health, Education, and Welfare, National Institutes of Health, National Cancer Institute grants CA-18628, CA-16480, CA-16672 and CA-17364.

Animals utilized in this study were maintained in facilities approved by the American Associated for Accreditation of Laboratory Animal Care, and in accordance with current United States Department of Agriculture and Department of Health, Education, and Welfare, National Institutes of Health regulations and standards.

### REFERENCES

BARLOGIE, B., DREWINKO, B., JOHNSTON, D. A., BUCHNER, T., HAUSS, W. H. & FREIREICH, E. J. (1976) Pulse Cytophotometric Analysis of Synchronized Cells *In vitro*. *Cancer Res.*, **36**, 1176.

BHAMBHANI, R., KUSPIRA, J. & GIBLAK, R. E. (1973) A Comparison of Cell Survival and Chromosome Damage using CHO Cells Synchronized with and without Colcemid. *Can. J. Genet. Cytol.*, **15**, 605.

BROWN, J. M. (1973) The Effect of Lung Irradiation on the Incidence of Pulmonary Metastases in Mice. *Br. J. Radiol.*, **46**, 613.

CRISSMAN, H. A. & TOBEY, R. A. (1974) Cell Cycle Analysis in 20 Minutes. *Science, N.Y.*, **184**, 1297.

FIDLER, I. J. (1973) The Relationship of Embolic Homogeneity, Number, Size, and Viability to the Incidence of Experimental Metastasis. *Eur. J. Cancer*, **9**, 223.

GLICK, D., VON REDLICH, D., JUHOS, E. & MCEWEN, C. R. (1971) Separation of Mast Cells by Centrifugal Elutriation. *Expl Cell Res.*, **65**, 23.

GRABSKE, R., LAKE, S., GLEDHILL, B. L. & MEISTRICH, M. (1975) Centrifugal Elutriation: Separation of Spermatogenic Cells on the Basis of Sedimentation Velocity, *J. Cell Physiol.*, **86**, 1977.

GRDINA, D. J., BASIC, I., GUZZINO, S. & MASON, K. A. (1976) Radiation Response of Cell Populations Irradiated *In situ* and Separated from a Fibrosarcoma. *Radiat. Res.*, **66**, 634.

GRDINA, D. J., BASIC, I., MASON, K. A. & WITHERS, H. R. (1975) Radiation Response of Clonogenic Cell Populations Separated from a Fibrosarcoma. *Radiat. Res.*, **63**, 483.

GRDINA, D. J., JONES, S., CHAN, E. & PETERS, L. J. (1977*a*) Influence of Cell Size and Cycle Parameters on the Development of Artificial Pulmonary Metastases. *Proc. 68th Ann. Meeting Am. Ass. Cancer Res.*, **18**, 67.

GRDINA, D. J., LINDE, S. & MASON, K. (1977*b*) Response of Selected Tumor Populations Separated from a Fibrosarcoma following Irradiation *In situ* with Fast Neutrons. *Br. J. Radiol.* (in press).

GRDINA, D. J., MEISTRICH, M. L. & WITHERS, H. R. (1974) Separation of Clonogenic Cells from Stationary Phase Cultures by Density Gradient Centrifugation. *Expl Cell Res.*, **85**, 15.

GRDINA, D. J., MILAS, L., HEWITT, R. R. & WITHERS, H. R. (1973) Buoyant Density Separation of Human Blood Cells in Renografin Gradients. *Expl Cell Res.*, **81**, 250.

HILL, R. P. & BUSH, R. S. (1969) A Lung Colony Assay to Determine the Radiosensitivity of the Cells of a Solid Tumor. *Int. J. Radiat. Biol.*, **15**, 435.

HITTELMAN, W. N. & RAO, P. N. (1974) Premature Chromosome Condensation: I. Visualization of X-Ray-Induced Chromosome Damage in Interphase Cells. *Mutation Res.*, **23**, 251.

HITTELMAN, W. N. & RAO, P. N. (1976) Premature Chromosome Condensation: Conformation Changes of Chromatin Associated with Phytohemagglutinin Stimulation of Peripheral Lymphocytes. *Expl Cell Res.*, **100**, 219.

HUMPHREY, R. & STEWARD, D. & SEDITA, B. (1970) DNA Strand Scission and Rejoining in Mammalian Cells. In: *Genetic Concepts and Neoplasia*. Baltimore: Williams and Wilkins, p. 570.

JOHNSTON, D. A., WHITE, R. A. & BARLOGIE, B. (1977) Automatic Processing and Interpretation of DNA Distributions: Comparison of Several Techniques. *Comp. Biomed. Res.* (Submitted).

MEISTRICH, M. L. (1977) Separation of Spermatogenic Cells and Nuclei from Rodent Testes. In: *Methods in Cell Biology*, Ed. D. M. Prescott. New York: Academic Press, p. 15.

MEISTRICH, M. L., GRDINA, D. J., MEYN, R. E. & BARLOGIE, B. (1977). Separation of Cells from Mouse Solid Tumours by Centrifugal Elutriation. *Cancer Res.*, **37**, (in press).

MILAS, L. & TOMLJANOVIC, M. (1971) Spleen Colony Forming Capacity of Bone Marrow From Mice Bearing Fibrosarcoma. *Eur. J. clin. Biol. Res.*, **16**, 462.

RAO, A. P. & RAO, P. N. (1976) The Cause of $G_2$-Arrest in Chinese Hamster Ovary Cells Treated with Anticancer Drugs. *J. natn. Cancer Inst.*, **57**, 1139.

STEINKAMP, J. A., FULWYLER, M. J., COULTER, J. R. HIEBERT, R. D., HORNEY J. L. & MULLANEY P. F. (1973) A New Multiparameter Separator for Microscopic Particles and Biological Cells *Rev. Scient. Instrum.*, **44**, 1301.

TOBEY, R. A. (1975) Different Drugs Arrest Cells at a Number of Distinct Stages in $G_2$. *Nature, Lond.*, **254**, 245.

WITHERS, H. R. & MILAS, L. (1973) Influence of Preirradiation of Lung on Development of Artificial Pulmonary Metastases of Fibrosarcoma in Mice. *Cancer Res.*, **33**, 1931.